# Leopard density and interspecific spatiotemporal interactions in a hyena‐dominated landscape

**DOI:** 10.1002/ece3.9365

**Published:** 2022-10-05

**Authors:** Sander Vissia, Julien Fattebert, Frank van Langevelde

**Affiliations:** ^1^ Wildlife Ecology and Conservation Group Wageningen University Wageningen The Netherlands; ^2^ School of Life Sciences, Westville Campus University of KwaZulu‐Natal Durban South Africa

**Keywords:** camera trap, *Crocuta crocuta*, *Panthera pardus*, *Parahyaena brunnea*, population density, spatial capture–recapture, spatiotemporal overlap

## Abstract

Scavenging is widespread in the carnivore guild and can greatly impact food web structures and population dynamics by either facilitation or suppression of sympatric carnivores. Due to habitat loss and fragmentation, carnivores are increasingly forced into close sympatry, possibly resulting in more interactions such as kleptoparasitism and competition. In this paper, we investigate the potential for these interactions when carnivore densities are high. A camera trap survey was conducted in central Tuli, Botswana, to examine leopard *Panthera pardus* densities and spatiotemporal activity patterns of leopard and its most important competitors' brown hyena *Parahyaena brunnea* and spotted hyena *Crocuta crocuta.* Spatial capture–recapture models estimated leopard population density to be 12.7 ± 3.2 leopard/100 km^2^, which is one of the highest leopard densities in Africa. Time‐to‐event analyses showed both brown hyena and spotted hyena were observed more frequently before and after a leopard observation than expected by chance. The high spatiotemporal overlap of both hyena species with leopard is possibly explained by leopard providing scavenging opportunities for brown hyena and spotted hyena. Our results suggest that central Tuli is a high‐density leopard area, despite possible intense kleptoparasitism and competition.

## Graphical abstract

Leopard densities were among the highest so far recorded for southern Africa and spatiotemporal interactions between leopard and their most important competitors, brown hyena and spotted hyena were intense.

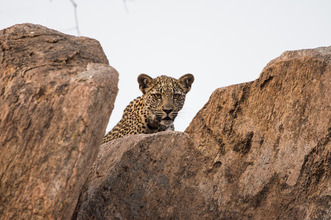

## 1 INTRODUCTION

Scavenging is widespread in the carnivore guild and has great implications for food web structure, population dynamics, and nutrient cycle (Barton et al., 2019; DeVault et al., 2003; Prugh & Sivy, 2020; Wenting et al., 2022). Interspecific interactions between carnivores can result in either facilitation or suppression of sympatric carnivores (Prugh & Sivy, 2020). Positive effects occur due to providing remainders of carcasses, whereas negative effects occur due to dominant predators killing subordinate predators or steal kills of other carnivores (Prugh & Ritland, 2005; Sivy et al., 2018; Van Dijk et al., 2008). These interactions can have a substantial impact on carnivore distributions and densities (Caro & Stoner, 2003; Linnell & Strand, 2000; Palomares & Caro, 1999; Ritchie & Johnson, 2009; Vissia et al., 2021). Carnivores are increasingly forced into close sympatry due to habitat loss and fragmentation, possibly resulting in more interactions such as kleptoparasitism and competition (Karanth et al., 2017). In this paper, we investigate the potential for these interactions when carnivore densities are high.

Due to the current worldwide decline in the distribution and population densities of large carnivores, changes in the structure and function of various ecosystem properties can occur (Ripple et al., 2014). Understanding the distribution and density of large carnivores is therefore vital to identify key conservation areas where source populations could persist at high density (Pitman et al., 2015) and to assess the effectiveness of conservation efforts (Blake & Hedges, 2004; Manning & Goldberg, 2010). In addition to estimating population densities, spatiotemporal activity patterns of sympatric carnivores can be evaluated (Burton et al., 2015) on which direct interactions such as kleptoparasitism can be derived. When kleptoparasitism occurs, we expect dominant and subordinate predators to co‐occur more at a certain location during a certain time span than expected by chance, as the species will meet at kill sites or because the dominant predator follows or harasses the other predator species (Cusack et al., 2017).

In this study, we used a camera trap survey in central Tuli, Botswana, to (1) accurately estimate the expected high density of leopard as subordinate, elusive carnivore species, and (2) quantify spatiotemporal interactions between leopard and its two most important competitors in the area, the brown hyena *Parahyaena brunnea* and spotted hyena *Crocuta crocuta*. The two hyena species have high densities compared with other locations (Vissia et al., 2021). Brown hyena and spotted hyena are known for stealing kills from leopard (Balme et al., 2017, 2019; Edwards et al., 2019; Stein et al., 2013), and we thus expected these three predators to co‐occur more frequently in space and time than expected by chance. We expected different patterns of co‐occurrence of these species for the dry and wet seasons as intraguild competition varies due to external conditions influencing resource availability (Owen‐smith & Mills, 2008; Vanak et al., 2013). During the dry season, a period of relative prey scarcity, competition between carnivores tends to increase (Vanak et al., 2013) while during the wet season when prey is more abundant (Pereira et al., 2014) competition might decrease. Consequently, we expected leopard, brown hyena, and spotted hyena to co‐occur more frequently in space and time during the dry season compared with the wet season.

## 2 MATERIALS AND METHODS

### 2.1 Study area

**FIGURE 1:**
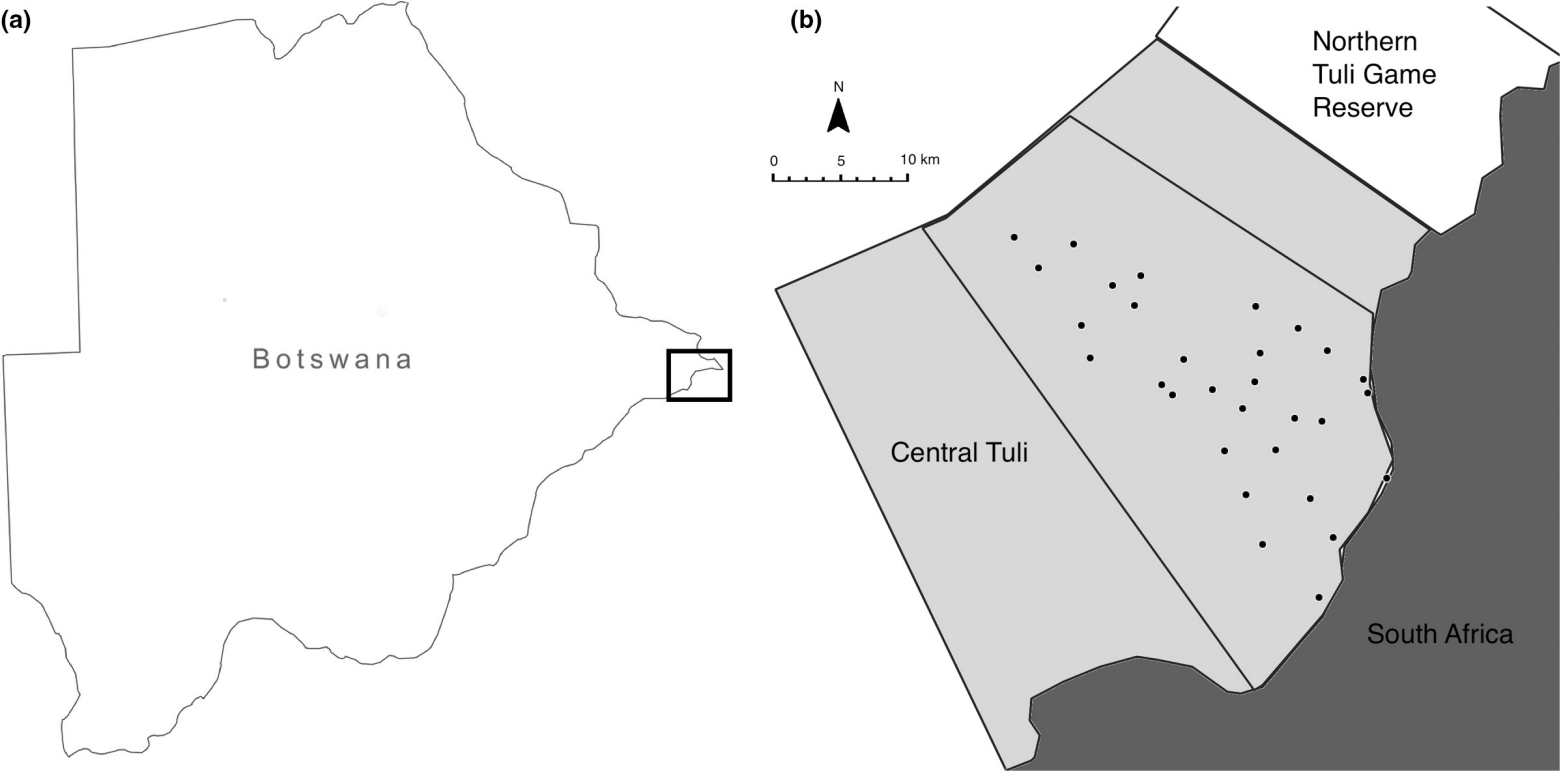
Map of (a) Botswana and (b) central Tuli (pale gray) including the location of the survey area (delineated area) and the camera trap stations for the leopard density estimation survey (black circles).

The study area is located in central Tuli, a protected area in South East Botswana of approximately 600 km^2^ (Figure 1). It is comprised of privately owned properties of which most host ecotourism lodges or private holiday houses and few properties have livestock with no fences between the individual properties (Vissia & van Langevelde, 2022). A 200 km^2^ area was delineated in central Tuli where a camera trap grid was used to sample for leopard and both hyena species.

The dominant flora is riverine woodlands with large bands of large fever berry trees (*Croton megalobotrys*) and mopane (Mopane‐Combretum) shrub savanna. Most precipitation falls during the wet summer months, spanning from November to April, with 350 mm average annual total rainfall. The carnivore guild consists of lion, leopard, spotted hyena, brown hyena, wild dog *Lycaon pictus*, aardwolf *Proteles cristata*, black‐backed jackal *Canis mesomelas*, bat‐eared fox *Otocyon megalotis*, African wildcat *Felis sylvestris lybica*, African civet *Civettictis civetta*, honey badger *Mellivora capensis*, and small‐spotted genet *Genetta genetta* all being present in the Tuli block (Vissia et al., 2021).

### 2.2 Sampling design and field methods

The study area was divided in different blocks (*n* = 3), and every block was sampled in rotation using a block‐survey design from November 2019 until February 2021 (Noss et al., 2013; Wang & MacDonald, 2009). An average of 11 camera trap stations was placed inside each block, whereby every camera trap station consisted of two camera traps and was placed at crossroads or game trails to maximize the capture probability of leopard and both hyena species (Vissia et al., 2021). Camera trap stations were spaced 1.5–2.5 km apart and placed on trees 2–3 m from the middle of the road at a height of 40–60 cm. Cameras were checked weekly to change batteries and download images. Cameras were set to run continuously and to take three photographs per trigger with a 5‐s delay between triggers with photo quality of 16 M‐pixels. Photographs that were recorded within 15 min of a previous photograph of the same species at the same camera trap station and could not be identified as a different individual were left out of the analysis as they cannot be considered an independent event (Kolowski & Forrester, 2017). Because two camera traps were placed at a station, both left and right flank photographs could be obtained for identification of leopard. Cubs <1‐year old were excluded from the analysis.

### 2.3 Leopard density estimation

We used camera trap data from the period November 2020–January 2021 to estimate leopard densities in a maximum‐likelihood spatially explicit capture–recapture framework with the R package “secr” (Efford, 2019). Capture histories were combined with each individual's location where it was detected. Additionally, “secr” produced two other parameters: the baseline encounter rate at the center of a home range g0, and *σ* describing how encounter rate decreases with increasing distance from the home range center. We fitted three a priori models to the data to estimate g0: (i) a null model, (ii) a learned response model (*b*, where leopard detection probability changes depending on previous captures), and (iii) a site learned response model (*b*_k_, where leopard detection probability changes at a particular site once it is caught on camera) (Thornton & Pekins, 2015).

A habitat mask was created to represent habitat that is potentially the activity center for each leopard individual of the population being studied. A buffer of the maximum mean distance moved (MMDM) was created using ArcGIS pro 2.4.2 (Esri, 2019) around the camera trap grid based on recommendations by Tobler and Powell (2013) and Sharma et al. (2010). Individual capture histories were constructed using 24‐h sampling occasions.

### 2.4 Temporal spacing of detections at shared camera trap station

To illuminate leopard, brown hyena, and spotted hyena activity patterns, package *overlap* in R was used (Meredith & Ridout, 2016) whereby each activity pattern was estimated separately using kernel density estimation (Meredith & Ridout, 2014; Ridout & Linkie, 2009). To test the hypothesis that the three predators co‐occur more frequently in space and time than expected by chance, we used time‐to‐event analyses to examine if leopards attracted its competitors (Balme et al., 2019; Cusack et al., 2017) using the data from the period November 2019–November 2020. In order to assess the importance of seasonality, the data were split into dry season and wet season. We recorded the number of hours separating the detection of leopard (reference detection) and the closest detection of the two hyena species at the same camera trap station in the 48 h before and after (proximal detection) since interactive processes were unlikely to occur over a longer time period (Balme et al., 2019). When the reference detection was followed by another detection of the same species, the latter detection was used as a new reference detection. The 48‐h time period was divided into eight 6‐h bins. For each bin, a detection probability was obtained by dividing the number of proximal detections falling into that bin by the total number of detections for the corresponding species (brown hyena and spotted hyena). To compare whether observed detection probabilities were more or less than expected if temporal spacing was random, we randomized the timing of proximal detections for a given species 1000 times to generate daily expected distributions following guidelines by Cusack et al. (2017). The expected detection probability values were compared with observed detection probabilities using standard permutation tests. Larger observed time‐to‐events than expected suggest species avoidance, while smaller observed time‐to‐events suggest species attraction (Balme et al., 2019).

## 3 RESULTS

We recorded 39 species of mammals, and other carnivore species captured were lion, wild dog, black‐backed jackal, bat‐eared fox, African wildcat, African civet, honeybadger, small‐spotted genet, and white‐tailed mongoose *Ichneumia albicauda*.

### 3.1 Density estimates

With a sampling effort of 1200 camera trap nights, a total of 88 independent leopard capture events were captured of which 73 (83%) could be positively identified. Leopard were captured at 25 camera trap stations (83%), and 20 leopards (7 different males and 13 different females) were captured on 55 sampling occasions. Individual leopards were captured on 1–8 occasions (mean = 2.75 ± 1.80). The best fitting spatially explicit capture–recapture model was the null model with an estimated leopard density of 12.7 leopard ±3.2 leopard per 100 km^2^, while the worst performing model was the learned response model with a very high standard error value (Table 1).

**TABLE 1:** Spatially explicit capture–recapture (SECR) leopard density estimates from three alternative models in central Tuli, Botswana, 2018–2021.

Model	*K*	Log likelihood	AICc	ΔAICc	*W*_*i*_	Density ± SE (leopard/100 km^2^)
*λ*_0_~1, *σ*~1 (null)	4	−148.0802	304.160	0.000	1	12.7 ± 3.18
*λ*_0_~*b*_k_, *σ*~1	5	−323.5327	657.065	354.524	0	12.2 ± 3.33
*λ*_0_~*b*, *σ*~1	4	−339.1625	688.325	385.784	0	23.88 ± 12.7

*Note*: Models were ranked according to their Akaike weights (*W*_*i*_) based on the Akaike information criterion for small samples (AICc). In addition to the null model, two models were fitted for g0 including a learned response model (*b*) and a site learned response model (*b*_k_).

### 3.2 Spatiotemporal co‐occurrence

**FIGURE 2:**
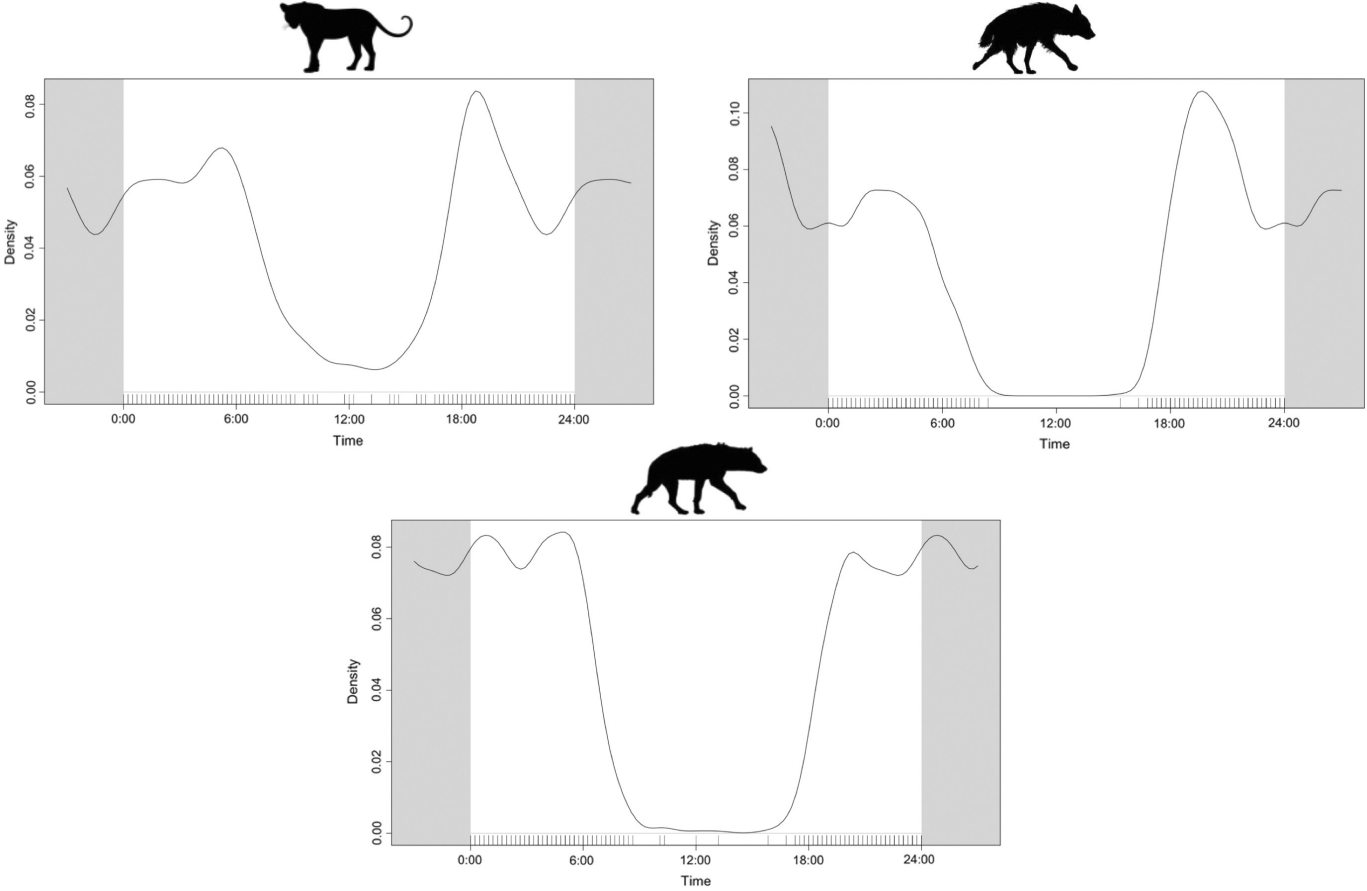
Kernel density estimates of the daily activity patterns of leopard, brown and spotted hyena in central Tuli, Botswana.

**FIGURE 3:**
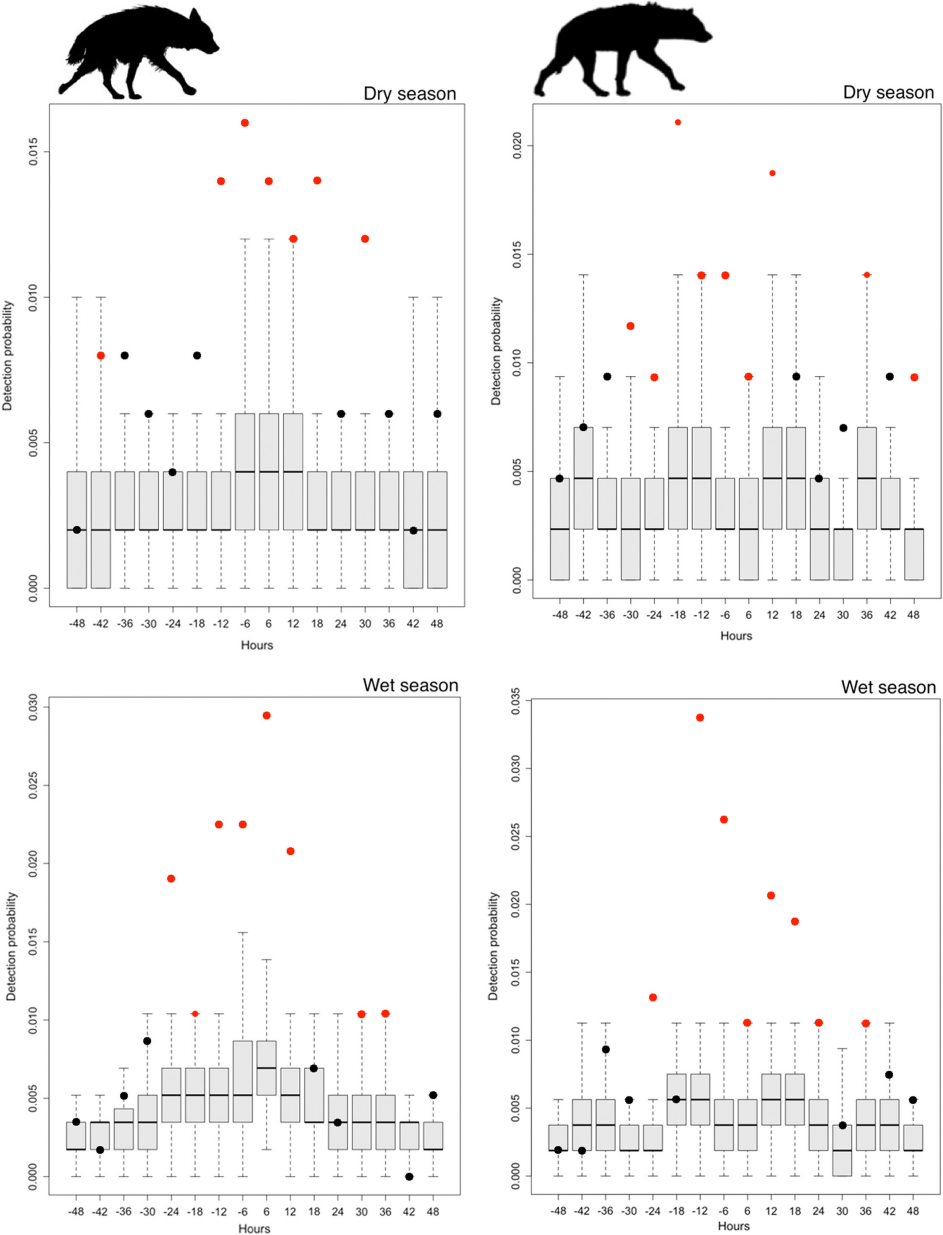
Detection probability of brown hyena (left) and spotted hyena (right) in the 48 h before and after a capture of leopard at the same camera trap station, divided into 6‐h bins in central Tuli, Botswana, in the period of September 2018–February 2021. Points indicate the observed detection probability of brown hyena and spotted hyena for each six‐hour bin before and after a leopard capture. Boxplots show the expected probability of detecting brown hyena or spotted hyena in each 6‐h bin before and after leopard capture. Expected detection probabilities were derived by randomly sampling 1000 times from the observed activity pattern probability density function for that species. Observed detection probabilities that differ significantly (*p* < .05) from the expected probability of detection are shown with red dots; those that do not differ significantly are shown with black dots.

Leopard, brown hyena, and spotted hyena all displayed crepuscular and nocturnal behavior (Figure 2). The time‐to‐event analyses showed that brown hyena were significantly more likely to be captured than expected in the hours before and after a leopard capture (*p* < .05 in all cases, Figure 3) in the dry season. Similarly, brown hyena were also significantly more likely to be captured in the hours before and after a leopard capture in the wet season.

In addition, spotted hyena were also significantly more likely to be captured in the hours before and after a leopard capture (Figure 3). This pattern was more marked in the wet season compared with the dry season. Since the simulated expected time‐to‐event distributions account for possible contrasting diel activity patterns between species (Cusack et al., 2017), these differences suggest the attraction of both brown hyena and spotted hyena by leopard.

## 4 DISCUSSION

In this paper, we assessed the potential for intraguild competition and kleptoparasitism when densities for all sympatric carnivores are high. We employed camera trap data to estimate leopard density and to reveal insights into interactive processes between leopard and their most important competitors, brown and spotted hyena, in central Tuli in Botswana. We estimated a density of 12.7 leopards/100 km^2^ in central Tuli, among the highest so far reported in Africa (Table 2). Similar or higher density estimates have been recorded in other locations, though contrary to our method these studies did not use camera trap data in a spatially explicit framework which may potentially lead to overestimating population densities (Noss et al., 2013; Tobler & Powell, 2013). Small sample area, low sampling effort, and using inaccurate and underestimated MMDM (buffer zone) can sometimes result in densities being overestimated by 200%–400%, or 3–5 times the actual density (Tobler & Powell, 2013). Multiple factors likely contribute to this high leopard population density in central Tuli: a combination of abundant prey, relatively low rates of human‐driven mortality and low lion density. The high leopard density occurs regardless the attraction of brown hyena and spotted hyena by leopard, whereas both hyena species are known to kleptoparasite prey from leopard (Balme et al., 2017, 2019; Edwards et al., 2019; Stein et al., 2013).

**TABLE 2:** Spatially explicit capture–recapture (SECR) leopard density estimates from previous camera trap surveys in Southern & Eastern Africa.

Location	Survey year	Density (ind./100 km^2^)	Reference
Timbavati Private Nature Reserve, South Africa	2013–2018	7.16–14.95	Rogan et al. (2019)
Karongwe Private Game Reserve, South Africa	2015, 2017	6.84–14.92	Rogan et al. (2019)
Okonjima Nature Reserve, Namibia	2015–2016	14.5	Noack et al. (2019)
Central Tuli, Botswana	2020–2021	12.70	This study
Mpala ranch, Kenya	2008	12.03	O'Brien and Kinnaird (2011)
Matusadona National Park, Zimbabwe	2013–2019	10.0–12.0	Loveridge et al. (2022)
iSimangaliso Wetland Park, South Africa	2014–2018	8.30–11.81	Rogan et al. (2019)
Sabi Sand Game Reserve, South Africa	2017	11.80	Balme et al. (2019)
Ithala Game Reserve, South Africa	2013–2018	8.43–11.1	Rogan et al. (2019)
Western Soutpansberg, South Africa	2008, 2012, 2016	3.65–10.70	Chase Grey et al. (2013), Williams et al. (2017)
Lajuma RC, South Africa	2014–2018	5.61–10.51	Rogan et al. (2019)
Makalali Game Reserve, South Africa	2014–2018	5.14–10.04	Rogan et al. (2019)
uMkhuze Game Reserve, South Africa	2013–2018	5.66–9.09	Rogan et al. (2019)
Pilanesberg National Park, South Africa	2016–2018	8.18–9.07	Rogan et al. (2019)
Munyawana Private Game Reserve, South Africa	2014, 2016–2017	6.66–8.71	Rogan et al. (2019)
Loskop Dam Nature Reserve, South Africa	2018	7.70	Morris et al. (2021)
Tembe Elephant Park. South Africa	2015–2018	5.15–8.38	Rogan et al. (2019)
Madikwe Game Reserve, South Africa	2017–2018	2.74–6.55	Rogan et al. (2019)
Venetia‐Limpopo Game Reserve, South Africa	2014–2017	4.86–6.52	Rogan et al. (2019)
Atherstone Game Reserve, South Africa	2013–2018	3.5–6.26	Rogan et al. (2019)
Lake Mburo National Park, Uganda	2018	6.31	Braczkowski et al. (2022)
Welgevonden Game Reserve, South Africa	2013–2018	2.24–6.07	Rogan et al. (2019)
Hluhluwe‐Imfolozi National Park, South Africa	2013–2018	3.28–6.03	Rogan et al. (2019)
Lapalala Wilderness, South Africa	2016–2018	4.33–5.60	Rogan et al. (2019)
Mangwe District, Zimbabwe	2010	5.12	Grant (2012)
Zambezi National Park, Zimbabwe	2013–2019	4.0–5.0	Loveridge et al. (2022)
Zingela Nature Reserve, South Africa	2016–2018	1–5.08	Rogan et al. (2019)
Udzungwa Mountains, Tanzania	2013–2014	4.22	Havmøller et al. (2019)
Mana Pools National Park, Zimbabwe	2013–2019	3.0–4.0	Loveridge et al. (2022)
Hwange National Park, Zimbabwe	2013–2019	2.0–4.0	Loveridge et al. (2022)
Phinda Private Game Reserve, South Africa	2012	3.40	Braczkowski et al. (2016)
Kafue National Park, Zambia	2016	3.34	Vinks et al. (2021)
Matetsi SA, Zimbabwe	2013–2019	0.2–0.3	Loveridge et al. (2022)
Wonderkop Nature Reserve, South Africa	2013–2015	0.87–2.97	Rogan et al. (2019)
Somkhanda Game Reserve, South Africa	2014–2017	1.74–2.89	Rogan et al. (2019)
Xonghile Game Reserve, Mozambique	2012	2.60	Strampelli et al. (2020)
Niassa National Reserve, Mozambique	2008–2010	2.18	Jorge (2012)
Chete SA, Zimbabwe	2013–2019	2.0	Loveridge et al. (2022)
Boland Mountain Complex, South Africa	2010–2011	1.69	Amin et al. (2022)
Cederberg Mountains, South Africa	2017–2018	1.53–1.62	Müller et al. (2022)
Chizarira National Park, Zimbabwe	2013–2019	1.0	Loveridge et al. (2022)
Ngamo & Sikumi Forests, Zimbabwe	2013–2019	1.0	Loveridge et al. (2022)
Chirisa SA, Zimbabwe	2013–2019	1.0	Loveridge et al. (2022)

Indeed, central Tuli provides high abundance of small and medium‐sized ungulates as prey for leopard and high levels of cover that may be associated with increased leopard hunting success (Balme et al., 2007). Line transect data revealed substantial populations of preferred prey species such as impala, steenbok, and common duiker (S. Vissia, unpublished data). For example, impala density, which is the most abundant herbivore and main prey species for leopard in central Tuli (S. Vissia, in prep) was 32.9 per km^2^ (Vissia et al., 2021) and may explain the high density for leopard. These findings are corroborated by Noack et al. (2019) who attributed an unnaturally high prey abundance as one of the main drivers of high leopard population density in Okonjima Nature Reserve, Namibia. In addition, Strampelli et al. (2020) attributed low leopard density in Xonghile Game Reserve, Mozambique, to lower prey densities.

Secondly, low rates of human‐driven mortality can also explain the high densities of leopard in the area. Leopards are known to leave the study area moving into neighboring farmlands (S. Vissia, pers. obs.) and high mortality rates among carnivores due to humans can occur when carnivores range beyond reserve boundaries (Balme et al., 2009; Loveridge et al., 2007; Schwartz et al., 2006), possibly leading to a decline of the carnivore population within the protected area itself (i.e., the edge effect; Balme et al., 2010; Woodroffe & Ginsberg, 1998). Retaliatory killings of leopards outside central Tuli only occasionally occur (S.V., pers. Obs.), hereby possibly contributing to the high leopard density.

Furthermore, leopards are likely not suppressed by intraguild predation due to low densities of lion in central Tuli also contributing to the high estimated leopard densities. While there is conflicting evidence of the importance of top‐down effects of lions on leopard population densities, intraguild competition killing is widespread among carnivores (Donadio & Buskirk, 2006; Palomares & Caro, 1999). Lions are known to kill adult leopards and leopard cubs, thereby decreasing reproductive success, but effects on population levels are not shown (Balme et al., 2013, 2017). However, Vanak et al. (2013) found that leopard had fine‐scaled avoidance behaviors and restricted resource acquisition tactics in the presence of lion.

Our spatiotemporal analyses showed that spotted hyena were captured more frequently following a leopard capture, findings supported by Balme et al. (2019) and Searle et al. (2021) and are possibly explained by the benefits of high spatiotemporal overlap of spotted hyena with leopard to increase kleptoparasitism and scavenging opportunities for the former species (Davis et al., 2021). Leopards are known to lose kills to spotted hyena, and high levels of kleptoparasitism can lead to lower reproductive success in female leopard (Balme et al., 2017) and therefore represents a threat to leopard fitness (Searle et al., 2021). In areas where population densities are high, spotted hyena might therefore have a negative impact on leopard. Despite our expectations, our results did not show seasonal differences and spotted hyena consequently were significantly more likely to be captured in the hours before and after a leopard capture both in the dry and wet seasons.

While several studies have looked at the impact of spotted hyena on leopard space use (Balme et al., 2019; Davis et al., 2021), research on spatiotemporal interactions between brown hyena and leopard is lacking. Similar to spotted hyena, brown hyena were more frequently captured before and after a leopard detection capture and no difference between dry and wet seasons was found. Where brown hyena and leopard co‐occur, diet of both species is similar (Williams et al., 2018) and brown hyena benefit from high spatiotemporal overlap with leopard since brown hyena frequently scavenge at leopard kills (Edwards et al., 2019; Owens & Owens, 1978; Stein et al., 2013). We therefore expected leopards to exhibit strong spatial or temporal overlap with both brown and spotted hyena. Since brown hyena and spotted hyena are ubiquitous across central Tuli, the high densities of brown and spotted hyena (Vissia et al., 2021) might make it impossible for leopard to avoid both hyena species. This widespread distribution of brown hyena and spotted hyena across the landscape possibly explains the high spatial and temporal overlap between the sympatric carnivore species. As a result, behavioral adaptations, like relying more heavily on their ability to cache kills in trees to avoid kleptoparasitism (Balme et al., 2017), might be necessary for leopards to minimize the fitness consequences of coexistence with hyenas (Searle et al., 2021).

Despite the potentially high risk of kleptoparasitism by brown hyena and spotted hyena due to high spatiotemporal overlap, it did not appear to result in negative population‐level consequences for leopard. Areas which host high densities of leopard and sympatric large carnivores can therefore possibly function as high‐density leopard source populations, despite intense intraguild competition, and could contribute to the broader population in the region via dispersal, provided that connectivity is maintained (Fattebert, Balme, et al., 2015; Fattebert, Robinson, et al., 2015; Pitman et al., 2017).

## AUTHOR CONTRIBUTIONS

**Sander Vissia:** Conceptualization (equal); data curation (equal); formal analysis (equal); funding acquisition (equal); investigation (equal); methodology (equal); project administration (equal); resources (equal); software (equal); supervision (equal); validation (equal); visualization (equal); writing – original draft (equal); writing – review and editing (equal). **Julien Fattebert:** Supervision (supporting); writing – review and editing (equal). **Frank van Langevelde:** Conceptualization (equal); methodology (equal); supervision (lead); writing – original draft (equal); writing – review and editing (equal).

## Data Availability

The data that support the findings of this study are available from the corresponding author upon reasonable request.
